# Learnings from the evaluation of HERrespect: a factory-based intervention to prevent intimate partner and workplace violence against female garment workers in Bangladesh

**DOI:** 10.1080/16549716.2020.1868960

**Published:** 2021-01-21

**Authors:** Ruchira Tabassum Naved, Mahfuz Al Mamun, Kausar Parvin, Samantha Willan, Andrew Gibbs, Rachel Jewkes

**Affiliations:** a Health Systems and Population Studies Division, Icddr,b, Dhaka, Bangladesh; b Gender and Health Research Unit, South African Medical Research Council, Cape Town, South Africa

**Keywords:** Intimate partner violence, workplace violence, workplace intervention, female garment workers, Bangladesh

## Abstract

**Background**: Intimate partner violence (IPV) and workplace violence (WPV) against women are widespread globally, and we set out to establish whether an intervention on gender-transformative programming delivered to Bangladeshi garment factory workers could reduce women’s experience of IPV and WPV. We developed and tested an intervention, HERrespect and encountered considerable obstacles.

**Objective**: To describe the challenges in program implementation and evaluation in the factories and the serious implications that arose for the study outcomes.

**Methods**: HERrespect is a participatory intervention with mostly parallel group sessions for female and male workers and the management staff, designed to be delivered weekly in three hourly sessions, and supported by some factory-wide and limited community information campaigns. It was evaluated in a quasi-experimental study conducted in eight garment factories in and around Dhaka city, with a cohort of 800 women workers and 395 management staff who were followed for 24 months.

**Results**: The study was conducted in the ready-made garment industry with substantial power imbalances between buyers, factory management and workers. The factories were contacted through the buyers, and some factories had agreed to participate half-heartedly. Many did not make enough time available for optimal implementation. Thus, the sessions were shortened and spread out. The factories did not make all the group members available for sessions. Whilst agreeing to participate, some management undermined the research by warning workers against disclosing information that may harm the business, resulting in the endline data being unreliable.

**Conclusions**: Future research on IPV prevention in this sector is advised to: (1) Gain genuine management buy-in prior to starting activities; (2) implement an optimally intensive programme for the workers and management; (3) engage men from the female workers’ communities. WPV prevention will require a change in the structural violence of the just-in-time regime which contributes largely to WPV.

## Background

Intimate partner violence (IPV) and workplace violence (WPV) against women are highly prevalent globally, with 30% of women aged 15 and over globally having ever experienced physical and/or sexual IPV [1]. There are no comparable rates of WPV, however studies from multiple workplaces, including the health sector [2] and factories [3,4] highlight this as a major problem. Bangladesh reports one of the highest levels of IPV in the world with over half (54%) of ever-married women reporting lifetime physical and/or sexual IPV and 27% reporting this violence over the past 12 months [5].

Women’s economic dependence on men exacerbates the gender power imbalance driving IPV against women. However, the impact of women’s employment on IPV experiences is mixed. Women’s engagement in work can establish an independent income, increase their bargaining power and provide economic autonomy to transform relationships at home [6]. However, work is not always empowering for women, it may also expose women to WPV and exploitation, and in highly patriarchal societies backlash against women’s employment may escalate IPV [7,8]. This may explain the observation that the prevalence of physical and/or sexual IPV amongst income-earning women in Bangladesh in the past year is higher than the national rate (33% compared to 26% for other women) [5].

Several studies have shown that programmes combining gender and economic empowerment can reduce women’s risk of experiencing IPV [9–11]. The original research examined an intervention with gender empowerment training overlaid on microfinance (revolving loan) schemes, but since then there has been research on a range of different economic empowerment approaches, and these have often shown similar success. To date, however, there has been no research to establish whether women’s economic autonomy, gained through workplaces, combined with gender empowerment intervention elements, may provide women with protection against IPV. A recent systematic review identified six studies evaluating five interventions that sought to respond to IPV, but none of them looked at whether IPV could be prevented through workplace intervention [12].

Bangladesh is an important setting for such research given that women workers are more vulnerable to IPV. Women’s labour force participation rate was 36% in 2016–17 [13]. In total 15% of women who work are employed in the manufacturing sector, including the ready-made garment (RMG) sector. The RMG sector has been a major driver of economic growth and formal employment for women in Bangladesh [14], with approximately 80% of workers in it being women [15].

In order to investigate whether a gender-transformative intervention in a workplace could prevent WPV and IPV, we developed HERrespect and set out to demonstrate proof of concept in a quasi-experimental study with a control arm. The intervention was designed to include female and male garment workers and managers and sought to improve gender equity, improve communication skills, build skills in non-violent forms of management, and socially empower female workers. However, the researchers and implementing partner found that the sector was extremely difficult to work in and numerous challenges were encountered in implementing and evaluating the intervention. In this paper, we present insights into the challenges in intervention implementation and evaluation that stem from the setting in Bangladesh’s garment factories that had serious implications for the study outcomes.

## HERrespect intervention

HERrespect intervention was developed by Business for Social Responsibility (BSR) and the South African Medical Research Council (SAMRC) as part of the DFID-funded What Works to Prevent Violence against Women and Girls (VAWG) global programme. There was initially a formative research, conducted by icddr,b [8], and this was used to construct a theory of change for the intervention, which guided the intervention planning and development of the manual for the workshop sessions [16]. The intervention had two components, the larger part of it was implemented in factories, delivered by a local women-led non-profit organization, Change Associates Limited, and there was a very small community component, implemented by WE CAN. The intervention components in the workplace included:

I. Gender transformative participatory intervention: This was designed as an 18-h curriculum consisting of six 3-h modules intended to be delivered weekly and engender critical reflection on gender norms, VAWG, staff relations in the workplace, and develop communication skills. The session topics included communication skills; assertive responses; reflection and discussion of gender roles and norms and relationships; power; violence in relationships; stress and conflict management; factory policy analysis; goal setting and being a Change Maker. The curriculum was participatory, taking reference from the Stepping Stones [10,17]. Complementary modules were developed for female workers, male workers and factory management.

There were also an additional three 90 minute sessions designed to bring together the different groups to promote communication. They were to be attended by groups of 25 made up from 10 female workers who were positioned as change agents, 10 managers and 5 male workers.

II. Factory-wide activities and campaigns: Factory-wide activities/campaigns included tailor-made messages to raise awareness of IPV, IPV prevention and available support services and were disseminated through factories public announcement systems, posters and a flyer.
III. Factory policy review and development: A meeting with the Factory Well-being Committee was held every two months. This committee is responsible for supporting workers’ physical and mental health and ensuring workers’ access to their rights. The topics included: reviewing existing and developing new gender policies and mechanisms to prevent and address sexual harassment; designing and implementing factory-wide promotional activities/campaigns; reporting on progress and challenges to senior management on a regular basis; and designing and implementing sustainability plans.

The small community engagement component, implemented by WE CAN, complemented the workplace initiatives by targeting the members of one community where a large group of female workers of a selected intervention factory lived. These activities included a courtyard community meeting, some door-to-door awareness work and screening a video to community members, on topics similar to the factory-wide campaign.

## Design of the evaluation

The evaluation was designed to provide a proof of concept for the intervention using a two-arm quasi-experimental design, with four factories in the intervention arm and four in the control arm. The factories selected for the evaluation were located in and around Dhaka, Bangladesh. Factories were contacted through the buyers, who arranged individual meetings and a workshop with the factories they worked with. The participating factories are those who volunteered to be recruited into the study. In the meetings, the implementing partner explained that we prefer factories that did not have any recent interventions on IPV or WPV involving training. Two factories working with each of the four buyers joined the HERrespect study. The factories expressed strong preference for being either an intervention or control factory, and were recruited on that basis. Control factories were selected at a distance from the intervention factories to prevent potential diffusion of the intervention effect. Two of the intervention factories were from special industrial zones characterised by better working conditions; however, we had no control factories from these specialised zones. Details of the study design are presented elsewhere [16].

### Factory profile

The factories were fairly large with an average of 1,342 workers and 121 managers at the time of recruitment in intervention factories and 2,129 and 164 in the control factories. Among the workers, 70% in the intervention factories and 59% in the control factories were women. Workers’ rights in the factories were limited, only one control factory had a trade union and five factories had an anti-sexual harassment committee in operation.

### Research participants and surveys

For the evaluation, we recruited 100 randomly selected female workers per factory from a factory-provided list of workers, who were currently married, living with their husbands and had been working in the factory at least one year. The selected workers from the intervention factories were assigned to the group sessions. A second cohort comprised 50 management staff per factory. Managers in the intervention factories were assigned to receive the group sessions for managers. Both samples were interviewed at baseline and then again two years later. We also had male workers in the group sessions but we did not interview them.

The baseline survey was conducted between September and December 2016 and the endline, between September and November 2018. The questionnaire for workers asked about their past year experience of physical and sexual IPV, and whether they had either experienced or witnessed workplace violence in the past 4 weeks. There were also questions on socio-economic status, gender attitudes, household, and husband’s characteristics. The questionnaire for managers asked about gender attitudes, management style, knowledge of and attitudes to laws and policies and burn out of management staff [16].

All 800 female workers and 395 management staff were interviewed at baseline. Due to anticipated high turnover rates amongst the workers, the workers’ cohort was tracked over the phone bi-monthly. By endline, 32% of the workers had left their factory but some of the workers were still living in the study sites and were still approached for interviews. All the workers were interviewed outside the factory (i.e. either at their own home or neighbour’s home) ensuring privacy. We had not anticipated that there would be much turnover of the managers, but found that by the endline 36% of the factory managers had left the index factory.

### Data analysis

The workers successfully interviewed in the baseline and endline surveys were included in the analysis, whether or not they attended the intervention or left the factory. We assessed the balance between the arms by comparing intervention and control samples at baseline. Chi-square and t-tests were performed for categorical and continuous variables, respectively, to test whether there were differences in background characteristics. When differences were detected, the relevant variables were controlled for in subsequent analyses. The impact of HERrespect intervention on the outcomes of interest were assessed using risk ratios derived from binary regression analyses for binary outcome variables adjusting for baseline rates. Analyses were adjusted for the potential covariates associated with the outcomes of interest.

### Ethical considerations

The study received approval from the Institutional Review Board (IRB) of icddr,b (PR#16036) and the South African Medical Research Council Ethics Committee (PR# EC013-5/2016). Factory participation was based on consent of the factory management. Individual verbal consent was sought prior to interviewing a worker or a manager. Each interviewed female worker received 6.5 USD (BDT 500) at baseline and 8.5 USD (BDT 650) at endline surveys in compensation for their time.

## Challenges faced in the intervention implementation and research

### Fidelity to the intervention design

The pressure to reach production targets in the factories presented a considerable obstacle to delivering the HERrespect intervention as it was designed. It was intended to follow a workshop-style ideally with a week or so between sessions to allow for experiential learning and reflection. The sessions were designed as three-hour sessions as this gave enough time to have a warm up, a reflection on the previous session and cover a coherent set of new activities that built on each other and ended on the right note and a closing activity. Splitting such a session hampered the flow and continuity, while shortening the session left insufficient time to address the material properly and limited the critical reflections.

With many of the factory management ambivalent about the intervention, there was sometimes reluctance to make the time for it to be delivered optimally. Thus, in some factories management did not allow workers to attend for 3 hours for the first few sessions, and insisted that the intervention be adapted and implemented in 1–2-h sessions. This compromised the design of sessions, which had a carefully considered flow, and time for reflective discussion. In addition, sessions could only be delivered once a month and so there was high possibility for participants to have forgotten what was previously discussed. Session attendance was also an issue, which may have been partly due to the factory management not reducing workers’ productivity targets on the days when sessions were held. As a result, instead of the intervention being delivered over about 6 weeks, it was delivered over a 10-month period with about one session per month, and some months skipped due to production target pressures and long Eid holidays.

### Ethical issues: privacy of the questionnaires

In Putting Women First, the WHO recommendations for ethical considerations in researching violence against women [18], there is discussion about the wisdom of masking the violence focus of the research so that there is no backlash against women completing them, or staff administering them, or else pressure put on the women to conceal their exposure to violence. We made efforts to follow this recommendation and so the study was framed as a ‘Survey of factory work, management and female workers life experiences’ at the household and community level. Workers were told that the questionnaire would include questions sensitive in nature only at the point when informed consent was sought for the research and the survey.

However, despite these efforts at concealment, the intervention factory management became aware of the likely focus of the research when the intervention was presented and discussed with them. They worried about the possible implications of violence being disclosed in the research for their sustained work with a brand and requested to see the women’s questionnaires before the baseline survey and the researchers complied.

### Impact of disclosure on reporting

The concerns expressed and manner in which the intervention factories gained access to the questionnaire at baseline raised a strong possibility of there being factory-level action that might lead to disclosure bias. Further bias that was differential as the control factories did not have the questionnaire at baseline. Table 1 shows the prevalence of violence reported by study arm at baseline. For all measures, there was very substantially greater reporting from the control factories. Differences in the volume and timing of information shared with the two different arms may have contributed to lower reporting of violence in the intervention arm compared to the control arm at baseline.

**Table 1.** Comparison of violence reports by women at baseline by study arm

	Intervention		Control	
	Baseline		Baseline	
N	303		304	
Physical IPV, past 12 m, %	27.7		38.8	
Sexual IPV, past 12 m, %	30.7		52.6	
Severe physical and/or sexual IPV, past 12 m, %	33.3		55.6	
Experiencing or witnessing of any WPV, past 4 w, %	58.1		84.9	

Before endline, management in two of the control factories also requested to see the questionnaires and were shown them. After we collected the endline data the research team learned that in at least six of the eight factories (3 per arm) management held meetings with workers participating in the study and advised them not to report anything that ‘may lead to their firing, closure of the factory and ill-repute the industry’.

Through observation during stay in the factories, the study team learned that two of the control factories had the worst working environment and highest levels of workplace abuse. In these, the factory management demonstrated a markedly negative attitude regarding the survey and were least helpful. In one of these factories one female worker interviewed had a bruise on her face. She did not report any WPV during the interview, but when the interview was over and the interviewer asked her about the mark on her face she started crying. She told the interviewer that they are severely abused in the factory, but could not protest or even talk about it, as they feared losing their jobs. She had four children to feed, so she did not even tell her husband about the physical assault. This factory had the highest rates of IPV and WPV at baseline and greatest reduction at endline. The other factory had relatively low rates of violence at baseline and huge reductions at endline. These findings suggest that the data collected in this study may have been severely compromised by the very conditions in the factories that we were seeking to improve through the intervention. This may explain the overall decline in violence reported in both arms by endline, and the fact that the rate of decline in the control arm exceeded that in the intervention arm (Table 2).

**Table 2.** Baseline to endline differences in outcomes reported by women workers by study arm

	Intervention		Control		Adjusted RR^#^ (95% CI) (Control = Reference group)
	Baseline	Endline	Baseline	Endline	
N	303	297	304	295	
Physical IPV, past 12 m, %	27.7	24.6	38.8	26.8**	1.3 (1.0–1.6)*
Sexual IPV, past 12 m, %	30.7	22.6^a^	52.6	27.8***	1.4 (1.0 − 1.9)*
Severe physical and/or sexual IPV, past 12 m, %	33.3	28.9	55.6	30.9***	1.6 (1.2–1.9)***
N	303	242	304	262	
Experiencing or witnessing of any WPV, past 4 w, %	58.1	74.8	84.9	79.8***	1.4 (1.2–1.6)***

*P < 0.05, **P < 0.01, ***p < 0.001, ^#^Adjusted for age, education and duration of work, clustering

## Discussion and lessons learned

Our study had the objective of evaluating the impact of HERrespect on violence in the workplace and in homes among women working in the garment factories. We did not achieve this, because (1) we were not able to implement the intervention with sufficient fidelity to be able to confirm that it was HERrespect, as designed, that was implemented; and (2) secondly because of interference in the research by factory management.

### Structural violence of the ready-made garment industry

When we commenced the study the research team was insufficiently aware of the structural violence that pervades the ready-made garment industry. The pressure of work in the industry and competition for factory survival created an intolerable macro-working environment. The brands all operated a just-in-time (JIT) regime, whereby orders were placed at factories very close to the time of product delivery to the market and so the pressure to complete orders was immense. This stressed managers and workers alike [3,19]. It tested the motivational skills of many managers to their limits, especially in the context of a management workforce which was 50% illiterate, and led to managers falling back on basic tactics of metaphorically and sometimes physically beating workers into production [20]. This left little scope for the intervention to be delivered in working hours and challenged the validity of the interventions’ theory of change which focused on downstream factors rather than the structural context of the industry.

### Coerced participation

Brands were very sensitive to the political climate in many of their marketplaces that had begun to demand better working conditions for garment workers. As a result, they passed on the pressure to ‘do the right thing’ down to factories, including becoming involved in the intervention, without the enabling environment being created by the buyers, brands and global fashion industry.

Working through the buyers we were successful in recruiting factories despite sensitive nature of our study. When the buyers approached the factories the management at least of some factories may have found themselves in an invidious position whereby they needed to keep positive relationships with their buyers and brands in order to stay in work. Thus, despite concerns about image and reputational risk the factories agreed to participate in HERrespect.

The downside, however, was that the number of participating factories was small (only 8) and the factories self-selected themselves into intervention and control arms not allowing factory-level randomisation. Thus, arms were not balanced at baseline, as one would expect in a well-randomised trial. Important differences between the arms were revealed. It also resulted in bias -due to factories that were most uncompromising in their emphasis on productivity wanting to be in the control arm as the study then had no impact on the working day, except for managers’ interviews. This may have explained the higher levels of violence reported by women in these factories at baseline.

### Assumptions underlying the intervention design

A key component of the intervention theory of change was the assumption that women were economically empowered as they were employed and received salaries, and that by providing gender-transformative interventions, this would reduce their experiences of violence. However, not all the garment factory workers were economically empowered, which may have impacts on the theory of change of the intervention. There were two aspects which undermined women’s economic empowerment through work. First, women were not paid well and worked long hours, and many experienced forms of coercion and economic control from factories. One-fifth (21%), for instance, did not have a letter of appointment, and many experienced factory managers controlling their earnings [8,21]. Second, some female garment workers do not retain control of their earnings. The earnings were either forcefully taken by the husband or handed over by the workers to their husband or mother-in-law [8,21]. As such, a key pathway through which violence could change, was not in operation.

### Pilot studies are essential even for adaptations

The context of working in these garment factories was so complicated that we were not able to subject the intervention to a small pilot, prior to the quasi-experimental study. At the time our main focus was on the intervention’s content, and we felt confident that the exercises would work as they were adapted from a very well-established intervention, known well to both the developer at the SAMRC and Change Associates in Bangladesh, who had previously used it in Bangladesh, and it had been found to be acceptable in other contexts. With hindsight, this was a mistake as it was the complexity of the setting that posed the greatest challenge. Without a pilot study, we did not have adequate insight into this before proceeding with wider intervention testing.

### Intervention design needs to be context-specific

We remain uncertain whether the design of the intervention itself could have been effective even if implementation had been optimal. There is a question about whether women, in the highly patriarchal context of Bangladesh, can be sufficiently empowered through a gender-transformative intervention to influence their exposure to IPV. Although the intervention was reasonably long, it is possible that without involvement of the women’s husbands (and possibly also their in-laws) it would have been hard to meaningfully change power relations in the home. However, one study in rural Bangladesh achieved reductions in rural women’s exposure to IPV using a cash transfer and group-based behaviour change communication programme [22].

## Conclusion

Accessing garment factories for research has long been widely recognised as a huge challenge in Bangladesh and elsewhere [23]. Wariness of this industry about research is due to widespread fear of harm to the business [23,24]. This study was particularly challenging as it involved not only research, but also intervention on a very sensitive topic within the industry – violence.

This study is a classic example of what may go wrong in an impact evaluation. However, the lessons are invaluable for future research and intervention. Our experience of developing and testing this evidence-informed intervention in factories in Bangladesh has led us to believe that the trial results do not really constitute an evaluation of HERrespect. The intervention was designed following a considered theory of change and is based on well-established behaviour change methods. We would recommend it still be evaluated through future research after genuine management support for the intervention has been secured, and a pilot to assess the feasibility of delivering it in factories.

A conducive environment is absolutely necessary for optimal intervention delivery for behaviour change. Contacting factories through buyers may be interpreted as posing implicit or imagined threats of withdrawal of work to the factory management, and disallow creation of a conducive environment.

We recommend not to share questionnaires with the factories. We suggest careful investment of much more time to establish trust before gathering data so that management does not fear ‘outing’ through research processes.

WPV is a serious issue in the garment sector demanding attention of the industry, as well as programme developers and policymakers [3]. Our experience with this work suggests that structural changes to the operation of the industry are needed to reduce WPV. However, we also need to implement and evaluate WPV interventions in factory settings to reduce the immediate burden female workers experience.

Our results suggest that the current focus and dose of factory management programme was not effective, and a more intensive programme, including more conventional staff management and performance motivation skills, may be needed to achieve an effect. This clearly points to the need to do a great deal of initial work to gain genuine management buy-in before starting an intervention of this nature with workers. Indeed, it may be desirable to first run the intervention groups with some managers before introducing the whole programme so that the intervention is better understood and seen to be beneficial before the research and intervention is rolled out.

Future work needs to also consider how to actively engage buyers in the processes of modifying their buying approaches, as the JIT regime shapes the context in which factories operate and the huge pressure workers face, which is critical in driving WPV. Eliminating violence and abuse in garment factories is vital for the health and wellbeing of all factory workers, and this needs both factory-level interventions as well as changes to the global practices of the fashion industry, particularly the just-in-time regime.

## Paper context

Based on the evidence that combining programs with women’s economic empowerment can reduce IPV in the community, we attempted to evaluate whether high level of IPV and WPV against female garment workers in Bangladesh can be reduced through a factory-based gender-transformative intervention targeting the workers and management. Both the intervention and evaluation faced numerous challenges with adverse ramifications for the outcome. These experiences provide great learning opportunities regarding what may go wrong in such initiative.
